# Transcranial direct current stimulation (a-tCDS) after subacromial injections in patients with subacromial pain syndrome: a randomized controlled pilot study

**DOI:** 10.1186/s12891-021-04139-2

**Published:** 2021-03-11

**Authors:** Samuel Larrivée, Frédéric Balg, Guillaume Léonard, Sonia Bédard, Michel Tousignant, Patrick Boissy

**Affiliations:** 1 Research Center on Aging CIUSSS Estrie CHUS, Sherbrooke, QC Canada; 2 Department of Surgery, Division of Orthopedics, Faculty of Medicine and Health Sciences, Université de Sherbrooke, Sherbrooke, QC Canada; 3 Research Center CRCHUS, CIUSSS Estrie CHUS, Sherbrooke, QC Canada; 4 School of Rehabilitation, Faculty of Medicine and Health Sciences, Université de Sherbrooke, Sherbrooke, QC Canada

**Keywords:** Subacromial pain syndrome, Rotator cuff tendinitis, Subacromial bursitis, Shoulder activity, Accelerometry

## Abstract

**Background:**

Subacromial pain syndrome (SAPS) is a common complaint in orthopaedics. Subacromial corticosteroid injections (CSI) can relieve pain in the short term. Anodal transcranial direct current stimulation (a-tDCS) has been used for symptomatic pain relief in a variety of chronic pain conditions. The aim of this pilot study was to assess whether the application a-tDCS could enhance the symptomatic relief provided by CSI in patients affected by SAPS.

**Methods:**

Thirty-eight participants (18 to 65-year-old) suffering from SAPS were recruited to have a CSI and randomly allocated to receive, 1 weeks post CSI, real a-tDCS (r-tDCS), sham tDCS (s-tDCS) or no intervention (Control). Upper limb function was measured 1 week prior to the CSI, at the 2- and 4-week follow-ups using self-administered questionnaires and physical measures. Self-reported pain and activity during each day were logged by the participants using visual analog scales (VAS). Differences between groups were tested using repeated-measures ANOVAs.

**Results:**

Pain VAS and the Single Assessment Numeric Evaluation scale (SANE) showed significant improvement from baseline 2 weeks and 4 weeks after CSI in all groups (*p* < 0.05). There were no significant group X time interaction 2 weeks following tDCS treatment in any of the variables.

**Conclusion:**

All groups showed significant improvement in pain VAS and SANE scores following the CSI. One session of a-tDCS treatment 2 weeks following CSI did not result in any additive or potentializing effects when compared to a s-tDCS or a control group.

**Trial registration:**

ClinicalTrials.gov, NCT03967574. Registered 30 May 2019 - Retrospectively registered.

## Background

Subacromial pain syndrome (SAPS) is a frequent cause of shoulder pain and disability in the elective orthopaedic surgery practice [1, 2]. This disease, caused by a spectrum of pathology ranging from subacromial bursitis to rotator cuff tendinopathy, often leads to shoulder stiffness and weakness, and subsequent decrease in function, difficulty performing activities of daily living (ADL), work disability, and lower overall quality of life [3, 4]. Symptoms usually respond well to conservative interventions such non-steroidal anti-inflammatory medications (NSAID) and physical therapy (PT) to decrease pain, improve scapular control and maintain shoulder range of motion [5, 6]. Further pain relief can be obtained with subacromial corticosteroid injection (CSI) which acts by reducing the inflammatory response around the tendons.

Multiple meta-analyses have been conducted comparing the effect of CSI to placebo in patients with SAPS. Mixed results have been reported, but generally a mild to moderate reduction in pain can be expected in the short term (less than 6 weeks) [7–10]. When compared to other interventions, relative efficacy is equivocal. CSI are superior to NSAID [7], but multiple randomized controlled trials have shown them inferior to physiotherapy in the treatment of SAPS patients [11–13]. Newer types injectable products, such a Platelet-Rich-Plasma (PRP) and hyaluronic acid, have shown mixed results in comparison to CSI [14–18]. At this time however, a positive response to CSI, defined as a reduction in pain and improvement in function assessed by the patient, is still considered an important prognostic factor that can guide the surgeon regarding the suitability of bursectomy or acromioplasty, a surgical treatment for persistent SAPS [19].

Enhancing the effects of CSI with a second non-invasive intervention could improve its usefulness in the clinical practice. For example, physiotherapy following a CSI provides more improvement in SAPS symptoms than physiotherapy alone [12, 20]. Another intervention, anodal transcranial direct current stimulation (a-tDCS) has been gaining attention in the literature and clinical practice as a mean to reduce pain [21]. Briefly, a-tDCS involves the continuous application of a weak electric current over specific areas of scalp for a period of several minutes using an inexpensive battery-powered device [22]. When directed over the primary cortical motor area of the brain (M1), a-tDCS has been shown to increase the excitability of the neurons of this brain region [23]. a-tDCS is believed to modulate the activity of cortico-thalamic fibers, inhibiting abnormal thalamic activity associated with persistent pain [22]. The wide surface area covered by the tDCS anode might also activate other neural networks, compounding its effect on pain [22]. It has been successfully used to improve pain management in non-specific chronic pain [21], lower back pain [24], fibromyalgia [25], stroke [26], osteoarthritis [27], and post-op pain [28, 29]. The affordability of this new technology, coupled with its safe adverse event profile [21], make it an interesting approach for the treatment of pain such as is seen in SAPS.

Corticomotor excitability of the rotator cuff M1 representation in patients with chronic rotator cuff tendinopathy has been shown to be decreased compared to the contralateral/asymptomatic side [30]. Thus, a-tDCS could be a valuable add-on intervention to CSI for patients with subacromial pain syndrome, with each intervention targeting distinct pathophysiological features; the latter addressing the peripheral/musculoskeletal component and the former reversing the maladaptive plasticity of the central nervous system [31]. Somewhat supporting this view are the results of previous studies suggesting that tDCS could have a « priming » effect on other interventions, potentially enhancing their effect by a synergistic mechanism [24, 32].

Thus, the principal objective of this pilot study was to explore whether the application of a-tDCS could enhance the symptomatic relief provided by CSI in patients affected by SAPS. The secondary objective was to measure if this treatment would translate into increased shoulder use and activity in this sample.

## Methods

### Study design

Patients (18 to 65-year-old) suffering from SAPS and scheduled for a CSI were recruited from the orthopedic service at the Sherbrooke University Hospital Centre (CIUSS de l’Estrie - CHUS) between January 6th, 2015 and April 30th, 2016 and randomly allocated to receive at 2 weeks post CSI real a-tDCS (r-tDCS), sham tDCS (s-tDCS) or no intervention (Control). The protocol was approved by the local Research Ethics Board (CIUSSS de l’Estrie – CHUS) and all participants were required to sign an information and consent form prior to their inclusion in the study. The protocol followed CONSORT guidelines and has been retrospectively registered using original documents initially submitted for ethics approval (ClinicalTrials.gov Identifier: NCT03967574). Participants were first seen 1 week prior to the CSI, at which time they filled in multiple questionnaires, received a clinical examination, and were finally given an accelerometer to wear for 5 weeks. Instruction for home-based exercises, consisting of internal and external rotation against elastic band resistance (three sets of 10 repetitions per day) were also given on that initial visit as per the treating orthopedic surgeon’s usual protocol. They received the CSI 7 days following the initial assessment. Two-weeks following the CSI, participants were randomized into one of three groups with a ratio of 1:1:1. Block-randomization was performed by an independent researcher using computer-generated random numbers and sealed into sequential envelopes. Physicians and assessors involved in enrollment, data collection and analysis were kept blind to group assignment for r-tDCS and s-tDCS until data collection was completed. Participants of these groups were also blinded to group assignment, while participants in the control were aware that they would not receive any tDCS intervention. Participants were reassessed 2 weeks (just prior to tDCS treatment) and 4 weeks after the CSI. Outcome variables were patient self-reported questionnaires, physical examination measures, and upper extremity use measured by accelerometry data. The questionnaires included the Western Ontario Rotator Cuff index (WORC) as the primary outcome, the short version of the Disability of the Arm, Shoulder and Hand questionnaire (QuickDASH), a daily pain visual analog scale (pain VAS), daily activity visual analog scale (activity VAS) and the Single Assessment Numeric Evaluation scale (SANE). The physical examination consisted of shoulder range of motion and shoulder strength. The time schedule of the study treatments and assessments is summarized into Table 1.

**Table 1:** Study participants schedule

Timepoint	Treatment/Procedure	Outcomes assessed
First assessment	Project start Beginning of home exercises (all patients)	Demographic questionnaire Baecke physical activity index WORC (baseline) QuickDASH (baseline)
Pre-injection week	Daily home exercises	Accelerometer data collection Daily pain VAS Daily activity VAS Daily SANE
Injection visit	Subacromial corticosteroid injection (all patients)	WORC (pre-injection) QuickDASH (pre-injection) Physical examination (pre-injection) Accelerometer data uploaded into computer
Post-injection weeks 1 and 2	Daily home exercises	Accelerometer data collection Daily pain VAS Daily activity VAS Daily SANE
tDCS visit	Patients randomized: - r-tDCS - s-tDCS - No intervention (control)	WORC (pre-treatment) QuickDASH (pre-treatment) Physical examination (pre-treatment) Accelerometer data uploaded into computer
Post-injection weeks 3 and 4	Daily home exercises	Accelerometer data collection Daily pain VAS Daily activity VAS Daily SANE
Final visit		WORC QuickDASH` Physical examination Accelerometer data uploaded into computer

Table 1 shows the distribution of the different assessments and treatment of the study

*Abbreviations*: *tDCS* transcranial direct current stimulation, *r-tDCS* real tDCS, *s-tDCS* sham tDCS, *VAS* visual analog scale, *SANE* single assessment numeric evaluation, *WORC* Western Ontario rotator cuff index

### Participants

Patients affected by SAPS on at least one shoulder were prospectively recruited from the practice of a group of upper extremity upper orthopaedic surgeons, posters, and referrals from physiotherapy clinics and family physicians. Diagnosis of SAPS was made by an orthopaedic surgeon based on physical examination and clinical judgment. Inclusion criteria were at least 9 months of symptoms, presence of a painful arc of movement, and at least one positive impingement sign (either Neer and/or Hawkin’s sign). Excluded were any participant presenting clinical or radiographic signs of another shoulder condition (gleno-humeral or acromio-clavicular osteoarthritis, adhesive capsulitis, cervicobrachialgia, rheumatic arthritis), clinically determined large rotator cuff tears, previous shoulder fracture or surgery, and shoulder CSI in the last 3 months. Potential participants were also excluded if they presented any contra-indications to a-tDCS or transcranial magnetic stimulation (TMS): presence of an epileptic disorder, metallic implants in any region of the brain, presence of brain lesion (vascular, metabolic, traumatic, or neoplastic), consumption of medication decreasing the convulsion threshold, alcoholism, planned or ongoing pregnancy. Following trial commencement, inclusion criteria were amended to include participants receiving worker’s compensation in order to facilitate recruitment efforts. Participants not eligible or refusing tDCS were also included and allocated to the control group.

### Interventions

#### Corticosteroid injection

The CSI was injected into the subacromial space using anatomic landmarks on the posterior aspect of the shoulder by the same fellowship trained shoulder surgeon for all participants. It consisted of a mixture of 1 mL of 40 mg methylprednisolone acetate and 4 mL of 1% lidocaine and was injected using a 25 bore one-and-a-half-inch needle.

#### Anodal transcranial direct current stimulation

**Fig. 1:**
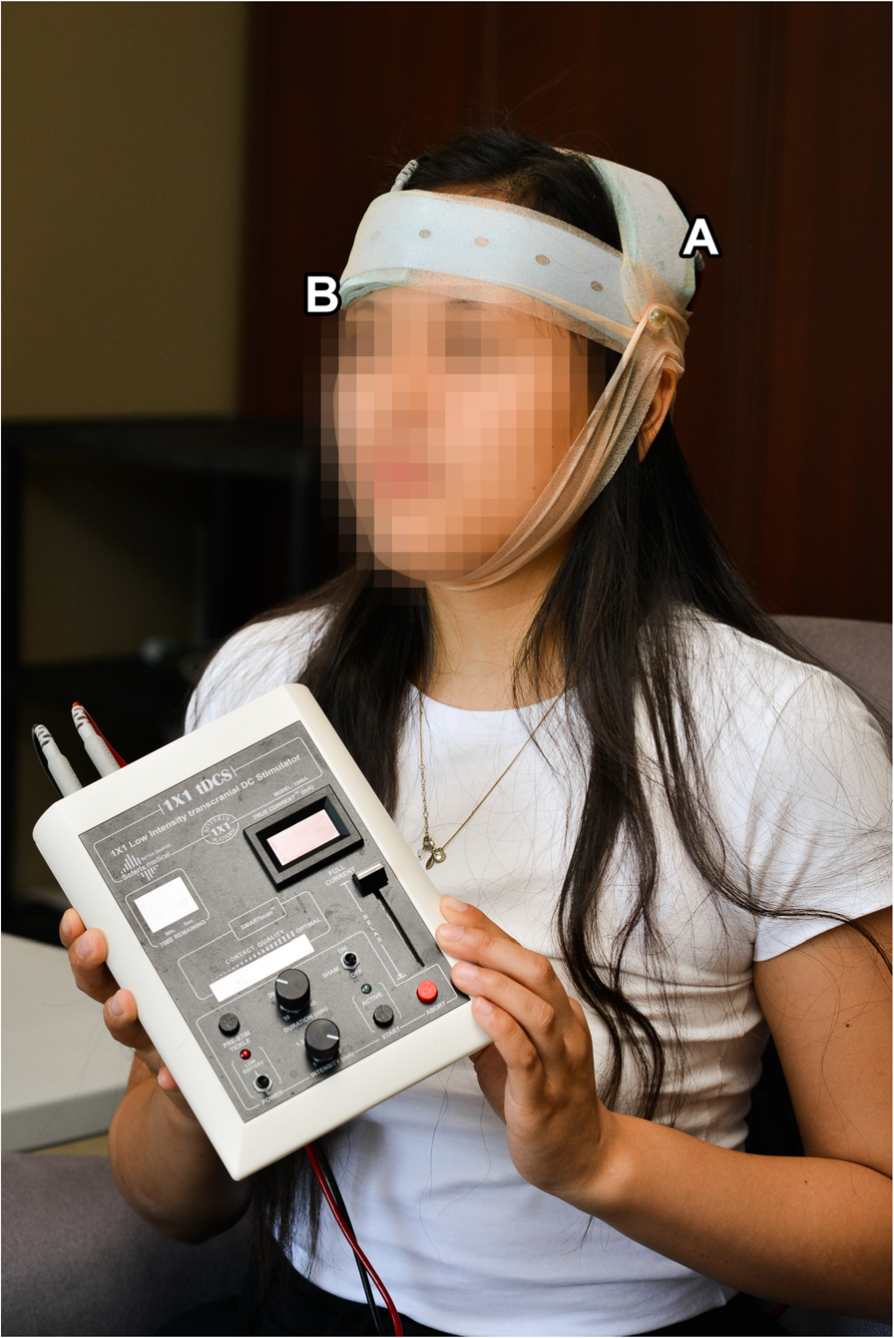
a-tDCS assembly. The anode (**a**) was applied on the M1 cortical zone for the rotator cuff as identified using TMS. The cathode (**b**) was applied over the contralateral eyebrow. The sponge electrodes where then secured with rubber band and adhesive tape and soaked in saline

The M1 zone for control of the affected rotator cuff tendons was first identified and marked using single pulse transcranial magnetic stimulation (TMS) and surface electromyography (EMG) according to the method previously described by Ngomo et al [30]. Two 5 × 7 cm tDCS sponge electrodes were soaked in 14 mL of saline solution to improve conductivity and used as anode and cathode. The anode was applied on the previously marked M1 cortical zone for the affected rotator cuff, while the cathode was applied above the eyebrow contralateral to the anode position, over the prefrontal cortex. The assembly was then secured with rubber bands and adhesive tape (Fig. 1). The study investigator then left the room while a research technician not involved in the assessment delivered r-tDCS or s-tDCS depending on the participant group assignation using a battery powered stimulator (Soterix Medical 1 × 1 tDCS device). The r-tDCS treatment was standardized for all participants and consisted of a continuous 2 mA stimulation for a duration of 20 min. The s-tDCS participants only received 30 s of stimulation at 2 mA followed by no stimulation the rest of the 20 min to mimic the initial tingling feeling of r-tDCS treatment. This protocol has been shown to adequately blind the participants in sham-controlled tDCS studies [33].

### Outcome variables

#### Questionnaires

The WORC and QuickDASH were both completed at four timepoints: just prior to the CSI injection, 2 weeks following CSI and prior to a-tDCS treatment, and 4 weeks following CSI. The WORC is a pathology-specific health-related quality of life questionnaire and has been well validated for the follow-up of rotator cuff disease [34]. The final score is reported on a scale from 0 to 100 points, with 100 points representing better function. The QuickDASH is a more general measure of upper extremity related quality of life and has been extensively validated [35–39]. Contrary to the WORC, a score of 100 indicates more severe disability. Daily questionnaires (pain VAS, activity VAS, and SANE) were also given to participants and filled for 1 week prior to the injection, and on the second and fourth week after. Each questionnaire was then averaged over the week measured. Additional baseline characteristics were recorded, such participant sex, age, dominant upper extremity, affected shoulder, time since onset of symptoms, Baecke physical activity index [40], and frequency of repetitive motion and overhead tasks at work.

#### Clinical examination

Patient’s shoulder strength and range of motion were assessed at each follow-up visit. Shoulder range of motion was measured with an inclinometer in three planes: abduction, flexion and flexion in the scapular plane (scaption). Shoulder rotator cuff strength was quantified with a portable dynamometer with three movements: abduction in scapular plane (Jobe maneuver), internal rotation with the arm at the side, and external rotation with the arm at the side.

#### Upper extremity Accelerometry

**Fig. 2:**
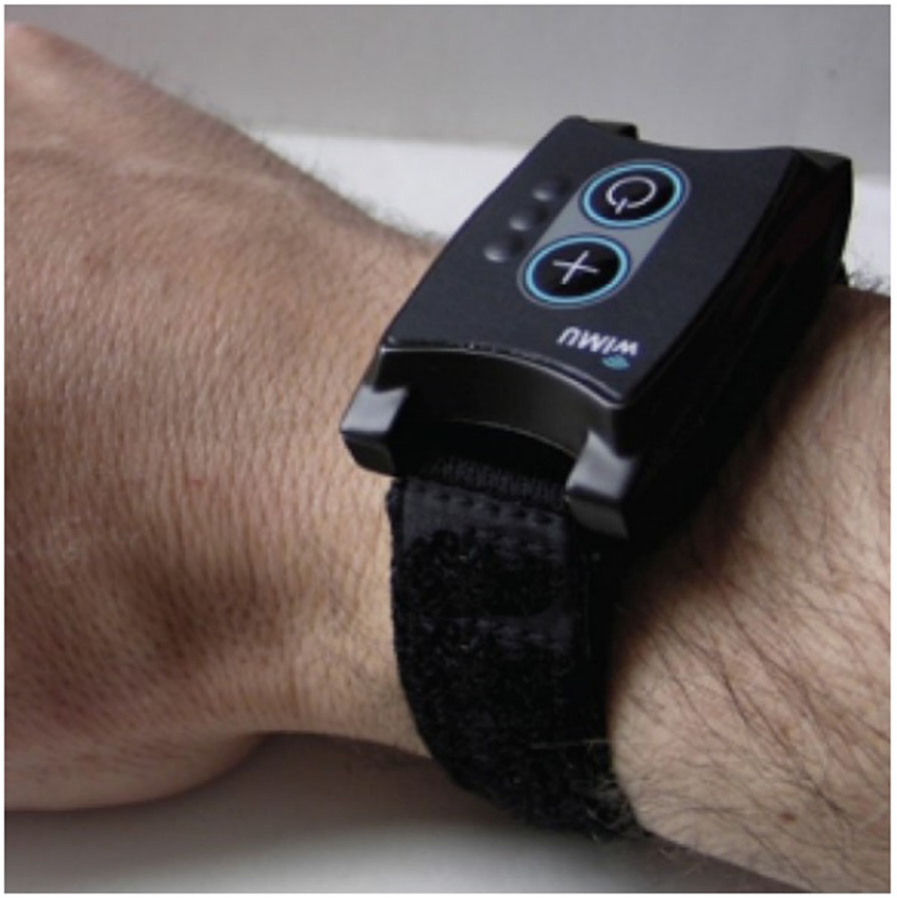
The WIMU-GPS accelerometer

A tri-axial accelerometer with a datalogger mounted in a wearable form factor similar to a wristwatch was used to quantify upper extremity daily activity (Fig. 2). The platform called WIMU-GPS (Wireless Inertial Measurement unit with GPS) was developed and assembled at the Research Centre on Aging to allow long term recordings of motion and location data from inertial sensors and GPS in a wearable form factor [41, 42]. The triaxial accelerometer (±2/4/8/16 g) was sampled at 50 Hz over the course of the day and the raw data was stored on a memory card. The WIMU-GPS was handed to the participants at the first evaluation and worn daily for a total duration of 5 weeks. They were required to wear the device at all time, except for water-based activity and at night for recharging. The accelerometer raw data was downloaded on a computer at each visit and processed as described in Larrivée, Balg [43]. Following low- and high-pass filtering (Butterworth, 1 Hz, 2nd Order; then Butterworth 5 Hz, 2nd Order), a unique acceleration vector was created using a square root sum calculation. A 10-s rolling window was then used to detect periods (or epochs) of activity, defined as epochs containing more than 50% of data points above a 0.015 g fixed threshold. The active time (AT) is obtained by summing the total time of detected activity over the day and dividing this by the total recorded time for that same day. Each epoch of active time was then integrated and summed for each day to create an activity count value. Daily activity count (AC) were defined as the mean activity count per minute of active time. Three other variables were obtained by separating the activity counts in three categories: low-intensity activities (LIA), moderate-intensity activities (MIA), and high-intensity activities (HIA). These were obtained by distributing the activity counts and defining LIA as activities below the 33rd percentile, MIA between the 33rd and 66th percentile, and HIA above the 66th percentile. These three variables are reported as a percentage of the total number of activities detected.

### Statistical analyses

Group and time interactions were tested using a repeated-measures ANOVA with Bonferroni correction for multiple comparisons. For variables collected over multiple days (daily activity and pain VAS, SANE, accelerometry variables), days were the patients had to present themselves to clinic removed to reduce observer effect and avoid collecting activity data on days that are not usually part of the participant’s routine. All daily variables were then averaged over 7 days to facilitate analysis and comparison with the other outcome measures. The SPSS statistics v22.0 software (IBM, Armonk) was used for the analysis, and the threshold for significance was set at 0.05 for bilateral testing.

As this pilot study was intended to gather data to be used to assess the feasibility of a larger randomized controlled trial, sample size calculations were not performed prior to commencement.

## Results

### Participants

**Fig. 3:**
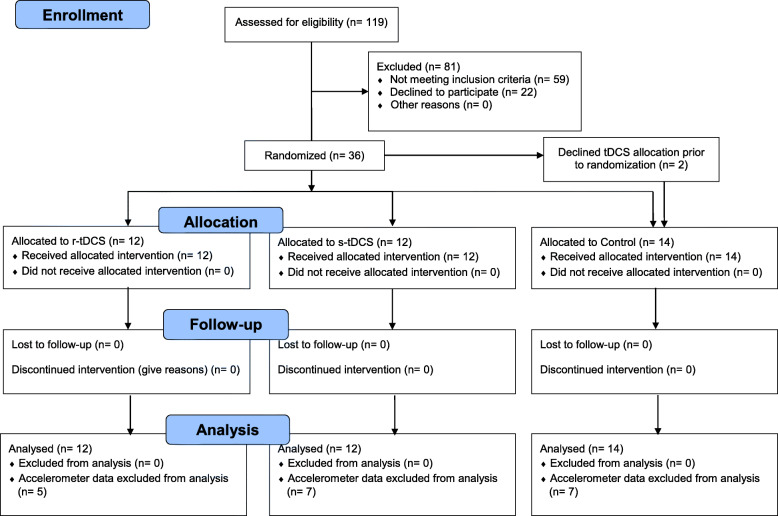
CONSORT flow diagram

Thirty-eight participants were recruited in this study: 12 received r-tDCS, 12 s-tDCS and 14 were allocated in the control group. Two participants refused to undergo TMS and tDCS and were placed in the control group (See Fig. 3, CONSORT Flow diagram). Participant’s characteristics of each group are summarized in Table 1. There were no significant differences between the three arms in baseline socio-demographic characteristics, questionnaire scores, strength, range of motion, or daily activity (See Tables 2 and 3). All participants were able to attend the pre-injection, injection, and four-week visits, but one participant was unavailable for the two-week assessment. The questionnaires for this patient were filled in and sent over by mail, but obviously no clinical examination could be performed at this endpoint. This participant was in the Control group and thus did not require tDCS treatment. Accelerometer data collected in the week preceding the injection was adequate for 24 participants, while they were sufficient for 22 at the second week following the injection and 19 at the fourth week.

**Table 2:** Baseline participants characteristics

Variable	r-tDCS group	s-tDCS group	Control	All groups
Number of participants	12	12	14	38
Sex	Male: 6 (50%)	Male: 7 (58.3%)	Male: 7 (50%)	Male: 20 (52.6%)
	Female: 6 (50%)	Female: 5 (41.7%)	Female: 7 (50%)	Female: 18 (47.4%)
Age (years ± SD)	52.0 ± 10.3 years	44.1 ± 6.9 years	50.2 ± 12.1 years	48.8 ± 10.4 years
Body mass index (BMI ± SD)	27.21 ± 5.0	28.7 ± 5.9	27.7 ± 4.36	27.9 ± 5.0
Dominant upper extremity	Right: 11 (91.7%)	Right: 10 (82.3%)	Right: 11 (78.6%)	Right: 32 (84.3%)
	Left: 1 (8.3%)	Left: 2 (16.7%)	Left: 3 (21.4%)	Left: 6 (15.7%)
Affected shoulder	Dominant: 9 (75%)	Dominant: 6 (50%)	Dominant: 6 (42.9%)	Dominant: 20 (52.6%)
	Non-dominant: 3 (25%)	Non-dominant: 6 (50%)	Non-dominant: 8 (57.1%)	Non-dominant: 18 (47.4%)
Time since onset of symptoms (months ± SD)	82.3 ± 75.3 months	88.6 ± 106.2 months	48.1 ± 50.3 months	71.4 ± 79.3 months
Baecke physical activity index (score ± SD)	8.7 ± 1.4	8.6 ± 1.9	9.1 ± 1.4	8.8 ± 1.5
Frequency of repetitive motions at work	Never: 1 (8.3%)	Never: 0 (0%)	Never: 0 (0%)	Never: 1 (2.6%)
	Rarely: 1 (8.3%)	Rarely: 1 (8.3%)	Rarely: 2 (14.3%)	Rarely: 4 (10.6%)
	Sometimes: 3 (25%)	Sometimes: 3 (25%)	Sometimes: 2 (14.3%)	Sometimes: 8 (21.1%)
	Often: 3 (25%)	Often: 4 (33.3%)	Often: 3 (21.4%)	Often: 10 (26.3%)
	Very often: 4 (33.3%)	Very often: 4 (33.3%)	Very often: 7 (50%)	Very often: 15 (39.5%)
Frequency of overhead tasks at work	Never: 2 (16.7%)	Never: 0 (0%)	Never: 0 (0%)	Never: 3 (5.3%)
	Rarely: 4 (33.3%)	Rarely: 4 (33.3%)	Rarely: 3 (21.4%)	Rarely: 11 (28.9%)
	Sometimes: 3 (25%)	Sometimes: 6 (50%)	Sometimes: 3 (21.4%)	Sometimes: 12 (31.6%)
	Often: 1 (8.3%)	Often: 1 (8.3%)	Often: 6 (42.9%)	Often: 8 (21.1%)
	Very often: 2 (16.7%)	Very often: 1 (8.3%)	Very often: 2 (14.3%)	Very often: 5 (13.2%)

Results shown as: total number (frequency %) unless otherwise noted

*Abbreviations*: *tDCS* transcranial direct current stimulation, *r-tDCS* real tDCS, *s-tDCS* sham tDCS, *SD* standard deviation

**Table 3:** Baseline participant scores

Outcome measure	r-tDCS group	s-tDCS group	Control	All groups
WORC	56.70 (17.69)	43.66 (22.20)	40.66 (15.96)	46.88 (18.86)
QuickDASH	27.58 (13.46)	44.43 (20.75)	42.41 (21.85)	42.85 (18.07)
Pain VAS^a^	48.26 (20.65)	53.58 (19.11)	61.84 (15.41)	54.94 (18.76)
Activity VAS^a^	59.02 (22.01)	55.78 (22.35)	59.69 (19.80)	58.24 (20.82)
SANE^a^	56.67 (17.68)	57.00 (16.40)	47.56 (19.05)	53.42 (17.92)
Strength (kg)				
*- Jobe*	7.82 (3.90)	8.09 (2.44)	7.92 (3.95)	8.11 (3.45)
*- External rotation*	8.56 (3.95)	9.57 (2.97)	9.23 (4.32)	9.27 (3.69)
*- Internal rotation*	13.27 (5.55)	13.29 (4.55)	13.60 (6.30)	13.60 (5.34)
Range of motion (°)				
*- Abduction*	160.83 (22.85)	164.33 (17.23)	160.14 (26.03)	161.86 (22.30)
*- Flexion*	157.92 (13.67)	163.50 (15.50)	155.64 (25.07)	159.49 (18.78)
*- Scaption*	159.75 (17.39)	170.18 (13.55)	159.14 (21.39)	163.75 (17.17)
Accelerometer				
- AT	0.5320 (0.1306)	0.5346 (0.0490)	0.4554 (0.1469)	0.50 (0.12)
- AC	317.19 (50.21)	329.61 (72.85)	293.06 (44.94)	309.75 (55.48)
- LIA	0.0567 (0.0260)	0.0490 (0.0140)	0.0646 (0.0217)	0.058 (0.021)
- MIA	0.2054 (0.0821)	0.2185 (0.1032)	0.2214 (0.0593)	0.217 (0.076)
- HIA	0.7609 (0.0973)	0.7531 (0.1122)	0.7367 (0.0714)	0.748 (0.088)

Results shown as Mean (SD)

*Abbreviations*: *SD* Standard Deviation, *AT* active time, *AC* activity count, *LIA* low intensity activity, *MIA* medium intensity activity, *HIA* high intensity activity, *VAS* visual analog scale

^a^Arithmetic mean for the first week

### Time and group interactions

Table 3 shows mean change in each variable according to group attribution. (See Table 4) Time X Group interactions were significant for the ratio of MIA (*p* = 0.005), ratio of HIA (*p* = 0.019), and pain VAS (*p* = 0.047), but not for any other variables. Pairwise comparisons with Bonferroni correction did not however yield any significant results.

**Table 4:** Effect of the injection at two and four weeks

Measure	Mean change (change SD) at 2 weeks			Mean change (change SD) at 4 weeks			Gr x time effect (p)
	r-tDCS	s-tDCS	Control	r-tDCS	s-tDCS	Control	
WORC	8.96 (17.61)	15.66 (17.41)	28.89 (23.37)	10.67 (17.51)	12.81 (16.88)	26.04 (27.41)	0.072
QuickDASH	−10.13 (22.56)	− 8.28 (12.88)	− 18.05 (16.24)	−8.64 (16.11)	− 7.61 (10.61)	− 14.68 (20.14)	0.366
Pain VAS^a^	−15.23 (12.40)	− 15.33 (20.06)	−28.82 (20.82)	−18.36 (20.13)	− 16.49 (14.83)	−35.81 (21.33)	0.047
Activity VAS^a^	6.20 (13.91)	0.87 (18.41)	13.57 (22.96)	4.57 (12.53)	4.67 (22.23)	11.02 (29.08)	0.430
SANE^a^	18.17 (18.21)	10.79 (15.10)	25.89 (18.82)	20.50 (20.20)	11.53 (12.62)	29.53 (18.98)	0.058
Strength (kg)							
*- Jobe*	−0.47 (3.52)	−1.42 (3.31)	−0.92 (2.44)	− 0.11 (3.45)	− 1.17 (2.96)	− 0.60 (2.66)	0.694
*- Ext rotation*	0.64 (2.10)	0.83 (1.45)	0.49 (1.58)	1.44 (2.57)	1.64 (1.16)	1.24 (2.42)	0.803
*- Int rotation*	0.69 (2.03)	0.76 (2.01)	0.44 (2.59)	1.68 (2.24)	1.89 (2.05)	0.64 (3.45)	0.178
ROM (°)							
*- Abduction*	−2.75 (14.55)	3.00 (16.03)	6.62 (17.25)	1.50 (18.94)	3.67 (13.49)	7.64 (15.64)	0.644
*- Flexion*	3.83 (18.85)	−0.33 (14.69)	3.31 (9.47)	0.75 (8.83)	0.17 (13.37)	8.14 (13.25)	0.626
*- Scaption*	−2.92 (15.61)	−1.36 (7.23)	3.31 (9.75)	2.58 (11.63)	−1.27 (7.27)	5.36 (13.04)	0.528
Accelerometer^a^							
- *AT*	−0.0653 (0.0382)	− 0.0235 (0.0517)	0.5339 (0.13)	−0.585 (0.0524)	0.0063 (0.0362)	0.0176 (0.1523)	0.159
*- AC*	−8.51 (14.81)	−23.81 (36.65)	−3.44 (40.60)	− 8.35 (63.45)	−31.02 (14.37)	−7.65 (25.18)	0.684
*- LIA*	0.0182 (0.0153)	0.0061 (0.0097)	−0.0149 (0.0289)	0.0255 (0.0302)	0.0142 (0.0053)	−0.0024 (0.0193)	0.338
*- MIA*	0.0363 (0.0141)	−0.0128 (0.0322)	0.0141 (0.1085)	0.0018 (0.0243)	0.0423 (0.0605)	0.0137 (0.0301)	0.005
*- HIA*	−0.0488 (0.0188)	0.0084 (0.0314)	−0.0007 (0.1240)	−0.0245 (0.0474)	− 0.0534 (0.0639)	−0.0100 (0.0387)	0.019

Results shown as Mean (SD)

*Abbreviations*: *SD* Standard Deviation, *Gr* Group, *Wk* week, *AT* active time, *AC* activity count, *LIA* low intensity activity, *MIA* medium intensity activity, *HIA* high intensity activity, *VAS* visual analog scale, *Ext* external, *Int* internal

^a^Change calculated from the average of the pre-injection week. and second and fourth week after the injection

### Adverse effects

No major adverse effects were reported by any patient in any of the study groups. However, some minor effects were spontaneously reported by participants within their daily logs. Four participants reported more shoulder pain following the initial clinical evaluation. Following CSI, three patients reported an increase in their shoulder pain, and one reported a headache. One participant from the s-tDCS group reported headaches after the sham tDCS intervention. All symptoms resolved within a week and did not require further medical evaluation.

## Discussion

Briefly, despite generally good improvement in all groups following CSI, no additive or potentializing effect of the tDCS treatment could be observed in this study, as there was no significant difference in any variables between the three groups.

At the end of the study, the sample recruited appeared to be representative of studied population. Similar to other studies on the effect of CSI [17, 44, 45], there is an almost even male to female distribution (52.6%: 47.4%) and the mean age is within the fourth or fifth decade. Alvarez, Litchfield [44] and Ekeberg, Bautz-Holter [45] report the dominant arm to be affected in about two third of the patients, while it was less so affected in the present sample (52.6%). Previous studies have shown that the difference in active time between the dominant and non-dominant upper extremity was only 30 min per day [46, 47]. As such, the dominance of the affected limb might play only a minimal role in the amount of shoulder movement detected from the wrist accelerometer and possibly contributing to daily symptoms. The long time between the onset of pain to the CSI (average of 71.4 months) might, however, have influenced the response to treatment of the injection, as the literature usually agrees that patients with SAPS respond better to conservative management when it is started earlier, usually within 6 months of the onset of symptoms [48–50]. This longer wait time is probably related to local and national factors secondary to this study being set in a publicly funded health-care system. Another Canadian study on SAPS also had significant wait times (45.6 months) from symptom onset to CSI [44]. Otherwise, the other participant factors (initial QuickDASH and WORC scores, BMI, smoking status, comorbidities and depression) were similar to previously published studies on SAPS. While not reaching statistical significance, distribution of baseline scores between groups shows trends toward important differences that might have affected final outcomes. Notably, and despite a thorough randomization process, baseline WORC and SANE scores were lower in the Control group than both r-tDCS and s-tDCS groups, while the inverse was true for QuickDASH and pain VAS (representing worse function or pain).

The only questionnaire showing a group and time interaction was the pain VAS. Despite not showing any significance after pairwise comparison and Bonferroni correction, there is a clear trend for the Control group showing larger decrease in pain than both tDCS groups. This is likely related to the higher pain scores seen at baseline for these participants, giving them a better potential for improvement. Randomization of the participants according to their baseline scores has been proposed to minimize this potential bias [51].

MIA and HIA showed significant group and time interaction but not significant after post-hoc Bonferroni tests. Trends show the MIA to increase and HIA to decrease in the r-tDCS group compared both s-tDCS and Control groups. Lack of significant improvement could be related to low sample size. Using sensitivity to change information from a previous publication of the present authors [43], a sample size between 22 and 70 patients would be necessary to be powered at 80% at 4 weeks, depending on the variable chosen. With only 19 accelerometers having valid data at baseline and 4 weeks, this threshold was not met. It is also conceivable that the subjective improvement in pain and function felt by the participants did not translate in a sizeable increase in their upper extremity activity. This hypothesis is supported by the fact that no statistically significant change in activity VAS was noted. This, however, contrasts some studies showing a significant change in kinematic scores after surgical repair of the rotator cuff [52–55]. Nevertheless, these studies differ significantly from the present study in terms of pathology, treatment and outcome variables, more than likely explaining the difference.

Additional explanations can be offered for the lack of effect of tDCS on pain. Firstly, the effects of tDCS on musculoskeletal pain are not well understood. While some studies with small number of participants have shown improvement in patients with non-specific chronic pain, lower back pain, and fibromyalgia [21, 24, 25], the well constructed trial by Luedtke, Rushton [56] was unable to show any additional effect of tDCS when done in conjunction with cognitive behavioral therapy for patients with low back pain. This study was a randomized double-blinded trial with a total of 135 participants (67 per group). Furthermore, the study by Belley, Mercier [57] explored the additive effects of tDCS and rehabilitation with sensorimotor training on patients with SAPS. Their trial was similar to the present trial in population studied and outcomes, and they were unable to detect any potentializing effect of the tDCS treatment either. It is also possible that the improvement shown with CSI may reach a ceiling effect. In contrast to earlier studies [12, 20], a 2016 randomized controlled trial did not show any additional affect of exercise therapy after a CSI [58].

The timing and dose of tDCS might also have played a role in the lack of tDCS effects. While this protocol only included 120 min treatment, recommended tDCS regimens usually consists of five daily treatments of 20–30 min each [59]. This regimen was chosen to reduce the number of participant visits, facilitate compliance to protocol, and document the effect of a single dose of tDCS as add-on to CSI. Fregni, Boggio [60] showed an additive effect of multiple sessions, with a clinically significant improvement compared to placebo only appreciable after three treatments. In a preliminary study on patients with lower back pain, significant improvements after a single session were only observed on experimental thermal pain thresholds but not on other aspects of pain [61]. Furthermore, tDCS might have had more effect on shoulder symptoms if it was provided before the CSI, as some studies have shown enhanced effects of subsequent treatment when tDCS is applied before, in contrast to after, the second intervention [32, 62]. It is also possible that a ceiling effect might have been reached after the CSI for most outcome measures, making it more difficult to detect additional improvements provided by the tDCS treatment. Finally, as most participants still had good relief at 4 weeks following the CSI, a longer follow-up period might have uncovered a prolonging effect of the tDCS treatment after 4 weeks.

Strengths of the study included strict inclusion criteria which ensured that only one pathology was followed and improving internal validity of the study. The follow-up rate was excellent, with no participant lost to follow-up. Utilisation of well validated questionnaires as well as more objective outcome measures such as accelerometry were also strengths of this protocol. The M1 area for the rotator cuff was localized using TMS in every participant, ensuring the tDCS treatment was delivered accurately at the intended target. The use of a randomized double-blind design also allows for increase internal validity.

However, some limitations must also be brought forward. Being a pilot study, the resulting of low number of participants may have explained the lack of power to detect a significant change between the groups. Using means and variance of the WORC score, this study achieved a power of 65.8%. The data from this pilot study can be used to determine that a minimum of 51 participants would be required to attain a power of 80% with an alpha set at 0.05. Despite strict inclusion criteria, no MRIs were performed to completely exclude other confounding pathologies. These strict inclusion criteria also reduce the external validity of the results. One of these criteria, the requirement of 9 months of symptoms prior to inclusion, also probably explains the large proportion of chronic SAPS in the sample. This choice was made to ensure that the SAPS would not self-resolve, as they can usually do within the first few months, and to align with referral criteria in place in the centre were this study was conducted. It is also worth noting that there are no gold standard clinical test to diagnose SAPS [63]. Randomization might have also been less successful than anticipated as the control group showed trends to worse baseline scores than the two other groups. The data was analysed on a “as treated” basis, in contrast to the “intention to treat” analysis that is recommend in randomized controlled trials. While no participants switched groups or assignation during the study, two patients who agreed to participate in the study declined to receive tDCS treatment. These patients were still included to increase the power of the study but may have biased the randomization process and the estimate of the efficacy of the intervention. Furthermore, the patients were only followed for 4 weeks after the CSI, and it is possible that a delayed effect might have been missed. However, a longer follow-up could have resulted in difficulty reaching target sample size and ensuring compliance with accelerometer wear. Some studies have also brought into question the adequateness of blinding sham tDCS in amplitudes of 2 mA [64]. Using only one tDCS session, provided after the intervention it is supposed to potentialize, might also explain the lack of effect, as explained previously. Finally, data loss from the accelerometer is a significant limitation of the study, decreasing the usability of this outcome measure in this study.

## Conclusions

One session of anodal tDCS treatment 2 weeks following CSI did not result in any additive or potentializing effects when compared to a sham tDCS or a Control group. Further studies should explore effects repeated tDCS sessions prior to the injection to improve the potentialization effect as well as interaction with other treatments such as physiotherapy.

## Data Availability

The datasets generated and analysed during the current study are available in the FigShare repository, https://figshare.com/s/f85915d26bf853988b44, DOI: 10.6084/m9.figshare.12331322. Raw accelerometer data is available from the corresponding author on reasonable request.

## Abbreviations

AC: Activity Count
ADL: Activities of Daily Living
AT: Active Time
CSI: Corticosteroid Injection
EMG: Electromyography
HIA: High Intensity Activity
LIA: Low Intensity Activity
MIA: Medium Intensity Activity
NSAID: Non-Steroidal Anti-Inflammatory Medication
PRP: Platelet-Rich Plasma
PT: Physical Therapy
QuickDASH: Short version of the Disability of the Arm, Shoulder and Hand questionnaire
SANE: Singe Assessment Numeric Evaluation Scale
SAPS: Subacromial Pain Syndrome
SD: Standard Deviation
tDCS: Transcranial Direct Current Stimulation
a-tDCS: Anodal tDCS
r-tDCS: Real a-tDCS
s-tDCS: sham tDCS
VAS: Visual Analog Scale
WIMU-GPS: Wireless Inertial Measurement unit with GPS
WORC: Western Ontario Rotator Cuff index
